# Smart Hydrogels for Bone Reconstruction via Modulating the Microenvironment

**DOI:** 10.34133/research.0089

**Published:** 2023-03-27

**Authors:** Weikai Chen, Hao Zhang, Qirong Zhou, Fengjin Zhou, Qin Zhang, Jiacan Su

**Affiliations:** ^1^ Institute of Translational Medicine, Shanghai University, Shanghai 200444, P. R. China.; ^2^ Organoid Research Center, Shanghai University, Shanghai 200444, P. R. China.; ^3^ National Center for Translational Medicine (Shanghai), Shanghai University Branch, Shanghai 200444, P. R. China.; ^4^ School of Medicine, Shanghai University, Shanghai 200444, P. R. China.; ^5^ School of Environmental and Chemical Engineering, Shanghai University, Shanghai 200444, P. R. China.; ^6^ Department of Orthopedics Trauma, Changhai Hospital, Naval Medical University, Shanghai 200433, P. R. China.; ^7^ Department of Orthopaedics, Honghui Hospital, Xi’an Jiao Tong University, Xi’an 710000, P. R. China.; ^8^ Department of Orthopaedics, Xinhua Hospital Affiliated to Shanghai Jiao Tong University School of Medicine, Shanghai 200092, P. R. China.

## Abstract

Rapid and effective repair of injured or diseased bone defects remains a major challenge due to shortages of implants. Smart hydrogels that respond to internal and external stimuli to achieve therapeutic actions in a spatially and temporally controlled manner have recently attracted much attention for bone therapy and regeneration. These hydrogels can be modified by introducing responsive moieties or embedding nanoparticles to increase their capacity for bone repair. Under specific stimuli, smart hydrogels can achieve variable, programmable, and controllable changes on demand to modulate the microenvironment for promoting bone healing. In this review, we highlight the advantages of smart hydrogels and summarize their materials, gelation methods, and properties. Then, we overview the recent advances in developing hydrogels that respond to biochemical signals, electromagnetic energy, and physical stimuli, including single, dual, and multiple types of stimuli, to enable physiological and pathological bone repair by modulating the microenvironment. Then, we discuss the current challenges and future perspectives regarding the clinical translation of smart hydrogels.

## Introduction

Bone defects caused by trauma, infection, malignancy, and osteoporotic fracture are prevalent with the lengthened lifespan and global aging trends [1,2]. Blood supply, age, and fundamental diseases, such as osteoporosis and diabetes, are variables that impact bone regeneration efficacy [3,4]. Among all clinically available grafts, autografts are the gold standard for treating bone defects. However, the limited supply, discomfort, and morbidity of the donor site, and the risk of wound infection restrict their use [5]. Allografts can overcome these problems, but there are still several challenges, including immune rejection, ethical controversies, unsatisfactory osseointegration, and disease transmission [6,7].

Tissue engineering has emerged as an attractive strategy for bone tissue reconstruction [8,9]. Extensive research has been conducted to engineer biomaterials, including various natural or synthetic polymers, for tissue engineering [9,10]. These biomaterials have the ability to modulate the extracellular microenvironment or drive cellular reprogramming to induce regeneration [11,12]. Notably, hydrogels have shown great potential in bone tissue engineering due to their unique advantages, such as good biocompatibility and biodegradability, tunable mechanical properties, excellent scalability, and injectability to fill irregular defects [13]. Moreover, hydrogels with a 3-dimensional (3D) network of polymers and a large percentage of water are similar to the native extracellular matrix (ECM) and provide excellent vehicles for bioactive molecules (e.g., growth factors, DNA, and small interfering RNA) and drug delivery and for cell encapsulation [14,15].

Smart hydrogels that can respond to internal and external stimuli in a spatially and temporally controlled manner have recently attracted much attention for bone therapy and regeneration [16]. Chemical and physical changes occur in these hydrogels or the embedded nanoparticles containing responsive moieties under the activation of specific stimuli (e.g., enzymes, reactive oxygen species [ROS], ultraviolet [UV] light, and temperature), allowing cargo (e.g., drugs, nanoparticles, and cells) encapsulation and release, ROS scavenging, oxygen production, and other processes [17–21]. The bone microenvironment is a highly dynamic system composed of multiple types of bone cells (such as osteoblasts, osteocytes, osteoclasts, and their precursors), hematopoietic cells, immune cells, stromal cells, adipocytes, fibroblasts, and endothelial cells, and the ECM with marked amounts of growth and signaling factors [22,23]. Compared to traditional hydrogels, smart hydrogels can achieve variable, programmable, and controllable changes on demand to modulate the bone microenvironment [24,25]. Remarkably, these hydrogels exhibit diagnostic and therapeutic functionality for treating pathological bone defects [26]. In addition to providing structural support, the smart hydrogels implanted into bone defects also activate osteogenesis- and immune-related signaling pathways, which regulate multiple cellular behaviors in the bone microenvironment, thereby promoting bone regeneration [27,28].

**Fig. 1.**
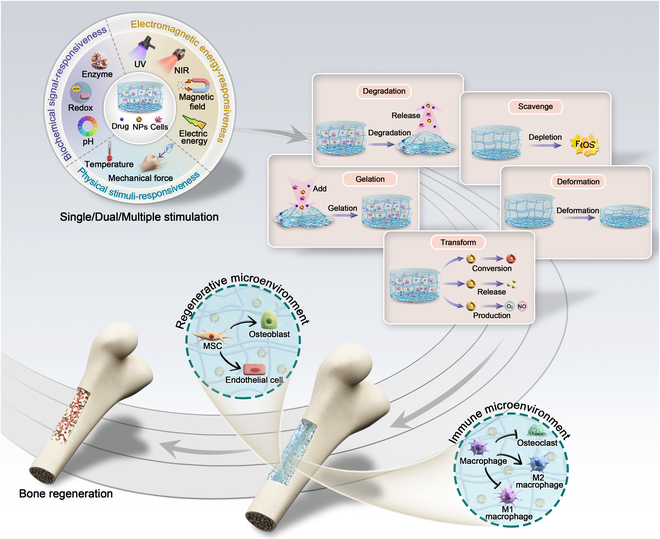
Schematic illustration of smart hydrogels applied to bone reconstruction by modulating the regenerative and immune microenvironment.

This review summarizes recent developments of smart hydrogels for bone regeneration with a focus on modulation of the bone microenvironment. First, we describe the advantages of smart hydrogels. Second, we summarize their polymers, gelation methods, and properties. Third, we describe the recent progress in hydrogels that respond to biochemical signals, electromagnetic energy, and physical stimuli, including single, dual, and multiple types of stimuli, to enable physiological and pathological bone repair (Table 1). In particular, we focus on bone regeneration directed by modulating the bone microenvironment using smart hydrogels. Finally, we discuss current challenges and future perspectives in this field (Fig. 1).

**Table.** Smart hydrogels for bone regeneration.

Types		Base hydrogels	Nanoparticles	Functional moieties	Features and advantages	Disadvantages
Biochemical signal	Enzyme	PEG [74]	/	MMP-sensitive peptides (GKKCGPQGIWGQCKKG)	The functional moieties are specific. The stimuli can induce degradation. The hydrogels can be used to encapsulate cells, carry exosomes, scavenge ROS, and deliver growth factors and drugs. It can regulate the local microenvironment and promote neurovascularization and bone regeneration.	Distribution and concentration of stimuli would change in pathological condition. The substrates could be recognized by similar enzyme families. Enzyme dysfunction affects the actions. Responsive behavior is uncontrollable. The responsive range of stimuli may be narrow. It may affect the surrounding tissue after implanting. The duration of the responsive behavior may be too short.
		PEG and DNA [56]	/	MMP-9 aptamer linker		
	Redox reaction	PEG [79]	/	Disulfide bonds		
		GelMA and m-PGA [88]	MnO_2_-coated calcium phosphate microspheres	/		
	pH	GelMA and OSA [93]	/	Imine bonds		
Physical	Temperature	Collagen and CA [94]	/	CA	These hydrogels possess reversibility, repeatability, and multi-interactions. They can carry exosomes, release ions, encapsulate cells, and deliver growth factors and drugs. The stimuli can induce hydrogel deformation and sol–gel phase transition.	The mechanical property of the network may not be sufficient. It may be affected by the surrounding temperature. The direction, intensity, and duration of mechanical stimuli should be explored.
	Mechanical	Ureido-pyrimidinone [103]	/	Multiple hydrogen bonds		
		Alginate [104]	PLGA microcapsules	/		
Electromagnetic radiation	UV	PEG [113]	/	*o*-Nitrophenyl group	The stimulus is noninvasive and safe, spatiotemporally controlled, precise, and has high tissue penetration. The stimuli can induce gelation and degradation, photothermal effects, drug release, and energy and signal conversion. They can monitor mineralization, deliver growth factors and drugs, and release nanoparticles and miRNA. These hydrogels provide outstanding regenerative effects without drugs or growth factors, promote M2 macrophage polarization, and induce endotheliocyte neogenesis and migration.	The parameters of the stimuli should be confirmed, such as intensity, time, range, and frequency. The equipment may be expensive and difficult to operate. The high local heat may be harmful to the surrounding tissue. Long-term biosafety and cytotoxicity should be confirmed. It may cause some side effects.
		GelMA and bisphosphonate [116]	/	Methacryloyl groups		
	NIR	MMA and GelMA [122]	Polydopamine nanoparticles	/		
		DCPH [123]	Calcium phosphate nanoparticles PNAm-ICG microspheres	/		
	Magnetic	PEG [127]	Superparamagnetic ions	/		
		Collagen fibers [129]	Superparamagnetic magnetic nanoparticles	/		
	Electro	Regenerate silk fibroin [52]	MXene nanosheets	/		

## Advantages of Smart Hydrogels

Hydrogels have a number of desirable qualities, such as biocompatibility, adaptable chemical and physical characteristics, modifiable composition, flexible manufacturing, and the capacity to replicate the ECM. Importantly, they have a 3-dimensional structure that can serve as a vehicle for drug delivery and cell encapsulation, thus attracting great interest in regenerative medicine. Hydrogels can be given various functionalities, such as injectability, elasticity, and adhesive properties, to meet a range of clinical demands, especially for bone repair. The synthesis techniques of hydrogels include physical (e.g., hydrogen bonds, electrostatic interactions, ionic contacts, hydrophobic interactions, and noncovalent connections) and chemical crosslinking (e.g., Michael additions, Schiff bases, and click chemistry) [29].

According to the response of hydrogels to stimuli, hydrogels can be divided into 2 categories: traditional hydrogels and smart hydrogels. Traditional hydrogels are not sensitive to environmental changes, while smart hydrogels can perceive stimuli in the internal and external environment (e.g., pH, enzymes, redox state, and temperature) and produce corresponding physical, structural, and chemical property changes [30]. The smart properties of these hydrogels are dependent on the functional moieties and nanoparticles in the hydrogel networks.

Compared with traditional hydrogels, smart hydrogels show substantial benefits in biological use. First, the sensitive moieties that initiate the phase transitions between the hydrogel and solution allow control of the gelation processes. When injected, for example, a thermo-sensitive hydrogel can transform from a solution into a gel due to the higher temperature. These automatically generated hydrogels may be useful while operating. Second, the chemical and physical characteristics of smart hydrogels after implantation can be altered in response to stimuli, which further modulate cells in the microenvironment. Furthermore, the use of smart hydrogels for drug delivery can reduce the dosage frequency, maintain the desired therapeutic concentration from a single dosage, and minimize drug side effects by preventing drug accumulation in nontarget tissues. Future treatment methods are being developed with a focus on microenvironment changes due to the rapid advancement of fundamental mechanistic research for bone-related illnesses. Therefore, smart hydrogels have good research and market application prospects because of their different responses in the bone microenvironment.

## Polymers for Smart Hydrogels

Smart hydrogels are the 3D networks of crosslinked hydrophilic polymers that can dramatically change their chemical and physical properties in response to environmental stimuli to achieve their biomedical application requirements [31,32]. Natural and synthetic polymers have been employed to create smart hydrogels. These polymers contain several reactive groups in their structure, which greatly enhance their ability to graft various functional groups, thus imparting versatile properties to these hydrogels. Through chemical or physical reactions, the polymers can be crosslinked to form hydrogels. The stimuli-responsive property of smart hydrogels is dependent on the responsive moieties that are included in the hydrogels or the embedded nanoparticles. In the following sections, we summarize the popular polymers used in smart hydrogels, including naturally derived polymers and synthetic polymers, while discussing methods of polymer modification and crosslinking to construct smart hydrogels.

### Natural polymers

Natural polymers derived from plants or animals, such as polysaccharides, proteins, and nucleic acids, have been widely used for the production of smart hydrogels because they are biocompatible, biodegradable, and nontoxic.

Alginate is an anionic polysaccharide that is obtained from brown algae [33,34]. Alginate-based hydrogels can be easily obtained by the addition of divalent or trivalent metal cations such as Ca^2+^, Mg^2+^, or Fe^3+^ as crosslinkers into an alginate solution to generate ionic inter-chain bridges under physical gelation [35]. In the physiological milieu, alginate hydrogel degradation is uncontrolled, and the molecular weight of released alginate strands is often higher than the renal clearance threshold. To control the degradation rate, oxidized alginate and cleavable crosslinkers have been applied in smart hydrogels, making them sensitive to specific stimuli [36,37].

Hyaluronic acid (HA), a major component in connective tissues, is the only nonsulfated glycosaminoglycan composed of repeated *N*-acetyl-d-glucosamine and d-glucuronic acid disaccharide units. Given that it contains free hydroxyl, carboxyl, and *N*-acetyl groups, HA can be easily modified with thiols, haloacetates, dihydrazides, aldehydes, or carbodiimide functional groups, enabling the crosslinking of HA hydrogels [38]. Chemical modification of HA with methacrylic anhydride provides an efficient strategy to form a smart hydrogel via photopolymerization [39].

Chitosan, a deacetylated product of chitin, is a linear polycationic polysaccharide composed of β-(1-4)-linked d-glucosamine and *N*-acetyl-d-glucosamine units with potent antimicrobial activity [40,41]. The numerous amine and hydroxyl groups in chitosan offer many opportunities for hydrogel formation via chemical crosslinking. Some reagents, such as glutaraldehyde, genipin, formaldehyde, and diacrylate, can react with these glucosamine groups to form chitosan hydrogels [42]. Furthermore, incorporation of chitosan with new functional groups that are favorable to a Schiff base reaction, disulfide bonding, or Michael-type additions allows for in situ smart hydrogel formation [43].

Collagen, a major fibrous protein in the ECM, provides tensile strength, supports cell adhesion, and directs tissue development. Although collagen exhibits an ideal natural polymer for biomedical applications, it requires time for collagen to self-assemble into a hydrogel [44]. Chemical modification of collagen with photo-crosslinkable functional groups, such as norbornene and methacrylic groups, enables smart hydrogel formation when exposed to visible or UV light [45].

Gelatin is a fibrous protein derived from the partial hydrolysis of collagen. Due to its reduced aromatic groups, gelatin has a lower immunogenicity than collagen [46]. The sol–gel transition temperature of gelatin is around 30 °C; hence, chemical crosslinking is necessary to prevent it from dissolving at body temperature. Photocrosslinkable gelatins are synthesized in the same manner as described above for collagen [47].

Silk fibroin is a natural protein extracted from *Bombyx mori* silk cocoons. It is a potential candidate for bone regeneration due to its excellent mechanical properties [48,49]. Under various treatments, including thermal, sonication, vortexing, pH, and alcohol treatments, a silk fibroin solution can be gelatinized [50]. To respond to specific stimulation, nanoparticles with responsive moieties are usually embedded into the smart hydrogels [51,52].

Deoxyribonucleic acid (DNA) is a polymer composed of 2 polynucleotide chains that contain genetic information [53]. DNA is an emerging material for bone repair because of its selective recognition and programmability. Watson–Crick base pairing via hydrogen bonding results in DNA strands forming a double helix shape [54]. DNA hydrogels with X-shaped, Y-shaped, and T-shaped DNA structures can be obtained [55]. In addition, DNA acts as a crosslinker to form smart hybrid hydrogels via hydrogen bonding [56].

### Synthetic polymers

Synthetic polymers such as polyethylene glycol (PEG), poly(vinyl alcohol) (PVA), poly(*N*-isopropylacrylamide) (PNIPAm), and poly(lactic-co-glycolic acid) (PLGA) have been used to form smart hydrogels that can respond to specific stimuli. Hydrogels made from synthetic polymers have better mechanical properties than those made from natural polymers. Additionally, synthetic polymers are unusually resistant to many chemical solvents during fabrication and processing without the concern of denaturation found in natural polymers. However, synthetic polymers have low bioactivity due to the absence of cell adhesion sites. Furthermore, the degradation of synthetic polymers is uncontrolled or slow. Chemical modifications are necessary to resolve these issues.

PEG has been approved by the Food and Drug Administration (FDA) for several medical applications [57,58]. PEG can be easily modified with various functional groups through its hydroxyl groups. Thus, it can form hydrogels through different mechanisms, including click chemistry [59], Michael addition reactions [60], light crosslinking [61], and Schiff reactions [62]. The matrix metalloproteinase (MMP)-cleavable peptides were used to crosslink a norbornene-modified PEG to create a UV-responsive and enzyme-responsive PEG hydrogel [63].

PVA, which is also approved by the FDA, is produced via partial or complete hydrolysis of polyvinyl acetate. The hydroxyl groups of PVA can be conjugated with various functional groups or peptides. PVA-based hydrogels can be formed via both physical and chemical crosslinking methods. Physical crosslinking of hydrogen bonding is used to create hydrogels with shape memory through a reversible process [64]. To enhance water stability of PVA, the free pendant carboxylic acid functional groups of citric acid were combined with the hydroxyl groups of PVA via an esterification process to create a pH-responsive hydrogel [65].

PNIPAm has been widely used to fabricate thermo-sensitive hydrogels due to its water solubility and tunable structures. It undergoes sol–gel phase transition at a temperature of approximately 32 °C, which is close to body temperature. PNIPAm usually acts as a backbone and combines with other polymers and nanoparticles to enhance osteogenesis [66,67].

PLGA has been approved by the FDA, and it is popularly used in biomedical applications. PLGA has superior biodegradability when compared to other synthetic polymers due to the hydrolysis of ester bonds and auto-catalytic degradation. The hydrophobic PLGA block is usually combined with the hydrophilic PEG block to prepare thermo-responsive hydrogels [68,69].

## Biochemical Signal-Responsive Hydrogels for Bone Regeneration

Numerous biological signal molecules are crucial for controlling cellular activity and tissue regeneration. Functional nanoparticles and functional polymers are used to create smart hydrogels that respond to biochemical signals, such as pH, enzymes, ROS, and temperature.

### Enzyme-responsive hydrogels

Tissue regeneration requires space for cells to migrate and adhere. Therefore, degradable biomaterials have been developed and found to be beneficial for promoting tissue healing. Enzyme-degradable hydrogels are an optimal choice due to their unique function and distribution. Common enzymes present in both normal and pathological bone metabolic processes include MMPs, azoreductases, phospholipidases, aggrecanase-1, and thrombin [70,71]. MMPs have a role in all stages of bone remodeling in the regenerative microenvironment, particularly in certain signaling pathways [72,73]. As a result, MMPs can be one of the triggers to induce the responsive behavior of hydrogels.

There are several methods to prepare hydrogels that respond to enzymes. One involves adding substrates to the hydrogels that are enzyme-susceptible. For example, amino acid sequences that are vulnerable to MMPs released by cells have been designed and used extensively. Due to the peptide thiol groups, MMP-sensitive peptide-based hydrogels are often created via click chemistry [36]. Li et al. [74] prepared a degradable PEG-based hydrogel containing MMP-cleavable peptides (GKKCGPQGIWGQCKKG) as a tissue-engineered periosteum (TEP). In the presence of lithium phenyl (2,4,6-trimethylbenzoyl) phosphinate (LAP) and under UV exposure, the norbornene groups of PEG were crosslinked with the thiol groups of peptides through a click reaction. Importantly, osteoprogenitor cells were encapsulated into the hydrogels to secrete MMPs to promote MMP-TEP degradation. Compared with hydrolytic hydrogels, MMP-degradable hydrogels further promote host cell infiltration, angiogenesis, and osteogenesis [75].

**Fig. 2.**
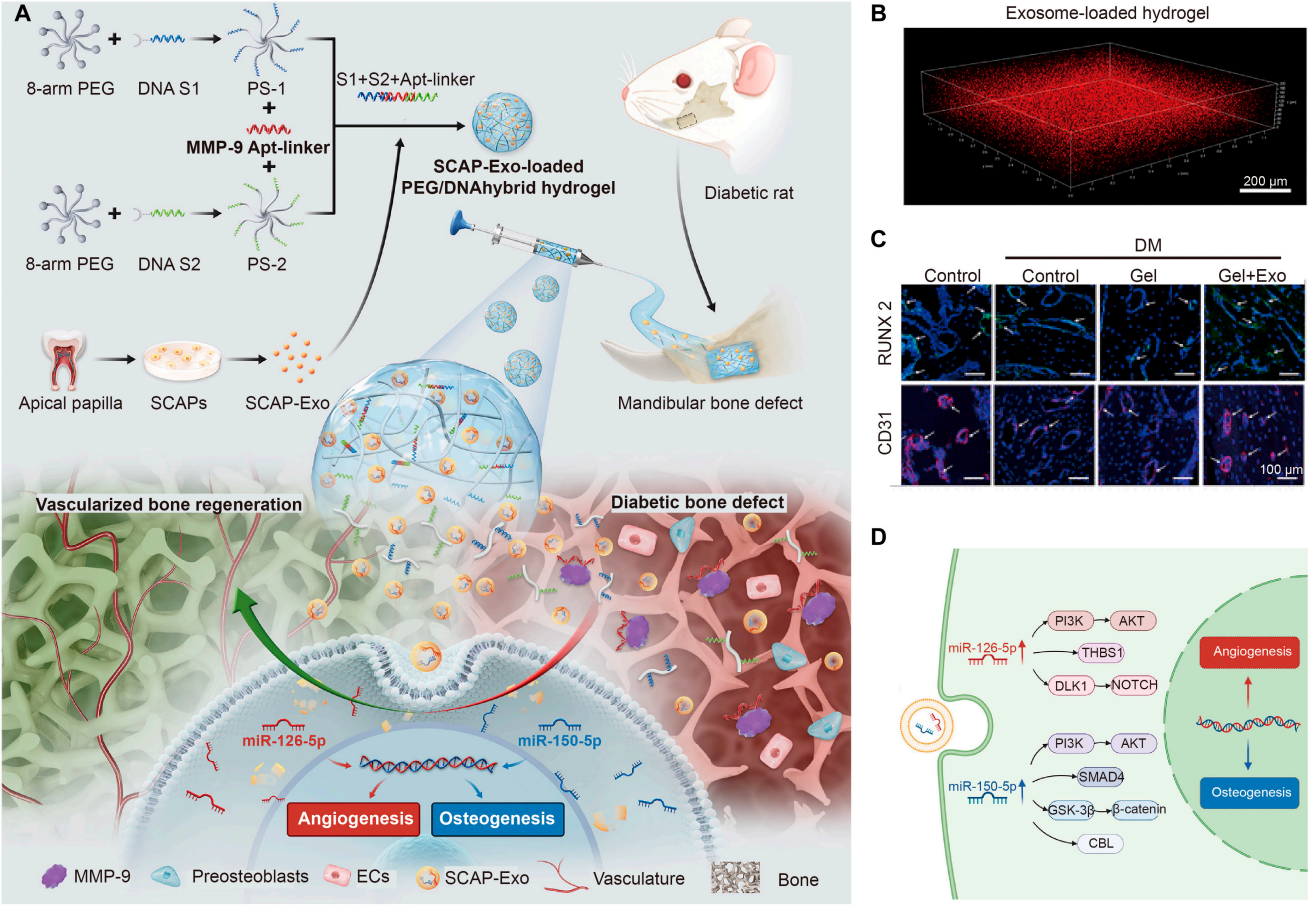
(A) Schematic illustration of hybrid DNA enzyme-responsive hydrogels for diabetic bone regeneration. (B) Exosomes dispersed homogeneously in the hydrogel. (C) Expression of *Runx2* and CD31 in the bone defect areas. (D) Signaling pathways of exosomes for angiogenesis and osteogenesis. Reproduced with permission from Ref. [56]. Copyright 2022 American Chemical Society.

The degradation of enzyme-responsive hydrogels not only provides space for tissue growth but also is beneficial for bioactive molecules and drug delivery. Compared with release triggered by hydrolysis in an uncontrolled manner, on-demand releases of growth factors, such as vascular endothelial growth factor, bone morphogenetic protein-2 (BMP-2), and basic fibroblast growth factor, would be more effective for bone repair. In pursuit of a sustained BMP-2 release system for osteogenesis, Schoonraad et al. modified BMP-2 with thiol groups and immobilized thiolated BMP-2 in an MMP degradable hydrogel via thiol-norbornene click chemistry. The BMP-2-tethered hydrogels degraded in response to MMP secreted from the neighboring cells, resulting in the triggered release of BMP-2 [76]. Thus, MMP-degradable hydrogels may be ideal vehicles for bone regeneration. Furthermore, intelligent MMP-degradable hydrogels can be further expanded by the use of aptamers that confer other advantages, such as a large range of targets, easy synthesis, and low cost [54,77]. For example, a DNA hybrid hydrogel incorporating exosomes was degraded by MMP-9, which was used to promote diabetic bone regeneration (Fig. 2A) [56]. Thiol-treated DNA strands interacted with vinyl sulfone functionalized PEG to create a hybrid DNA polymer. The hybrid DNA polymers were then crosslinked with MMP-9 aptamer linkers to generate smart hydrogels, in which MMP-9 in the bone microenvironment identified and degraded hydrogels to release the encapsulated exosomes (Fig. 2B). The release of exosomes significantly increased the expression of Runx2 and CD31 (Fig. 2C), as well as miRNAs (miR-126-5p and miR-150-5p), thereby promoting angiogenesis and osteogenesis in a bone regenerative microenvironment (Fig. 2D).

Therefore, the most impressive characteristics of enzyme-responsive hydrogels, which usually includes enzyme-sensitive substrates, are specificity and efficiency. However, the substrates may be recognized by similar enzyme families. Thus, novel peptides and aptamers could be developed to target and recognize the enzymes. Moreover, compared with the physiological microenvironment, the function, concentration, and distribution of enzymes may vary under pathological conditions. It may be feasible to identify and target stable enzymes in physiological and pathological microenvironments.

### Redox-responsive hydrogels

The redox-responsive hydrogels undergo oxidation–reduction reactions when exposed to the physiological microenvironment that contains superoxide, H_2_O_2_, and reductants. They can be divided into reduction-responsive hydrogels and oxidization-responsive hydrogels.

Generally, the reduction-responsive hydrogels are prepared by introducing reductive-labile linkers, such as disulfide bonds, or succinimide-thioether. Glutathione (GSH) secreted by local cells, a reducing agent, is commonly found in tissue regeneration microenvironments [78]. The reduction-responsive hydrogel is degraded where the host cells migrate and generate GSH. It is feasible to develop a degradable hydrogel by leveraging GSH. For example, Yang et al. reported a GSH-responsive PEG hydrogel as a drug delivery system to promote bone healing [79]. A thiol PEG (PEG-SH) was synthesized, and then, the PEG-SH precursor containing BMP-2 was mixed with H_2_O_2_ to form GSH-sensitive hydrogels through disulfide bonds. Depending on the in vitro GSH content, the backbone of the hydrogels degraded over 0.5 h to 22 days. Therefore, the degradation of hydrogels could provide proper space in the regenerative microenvironment for tissue regeneration.

**Fig. 3.**
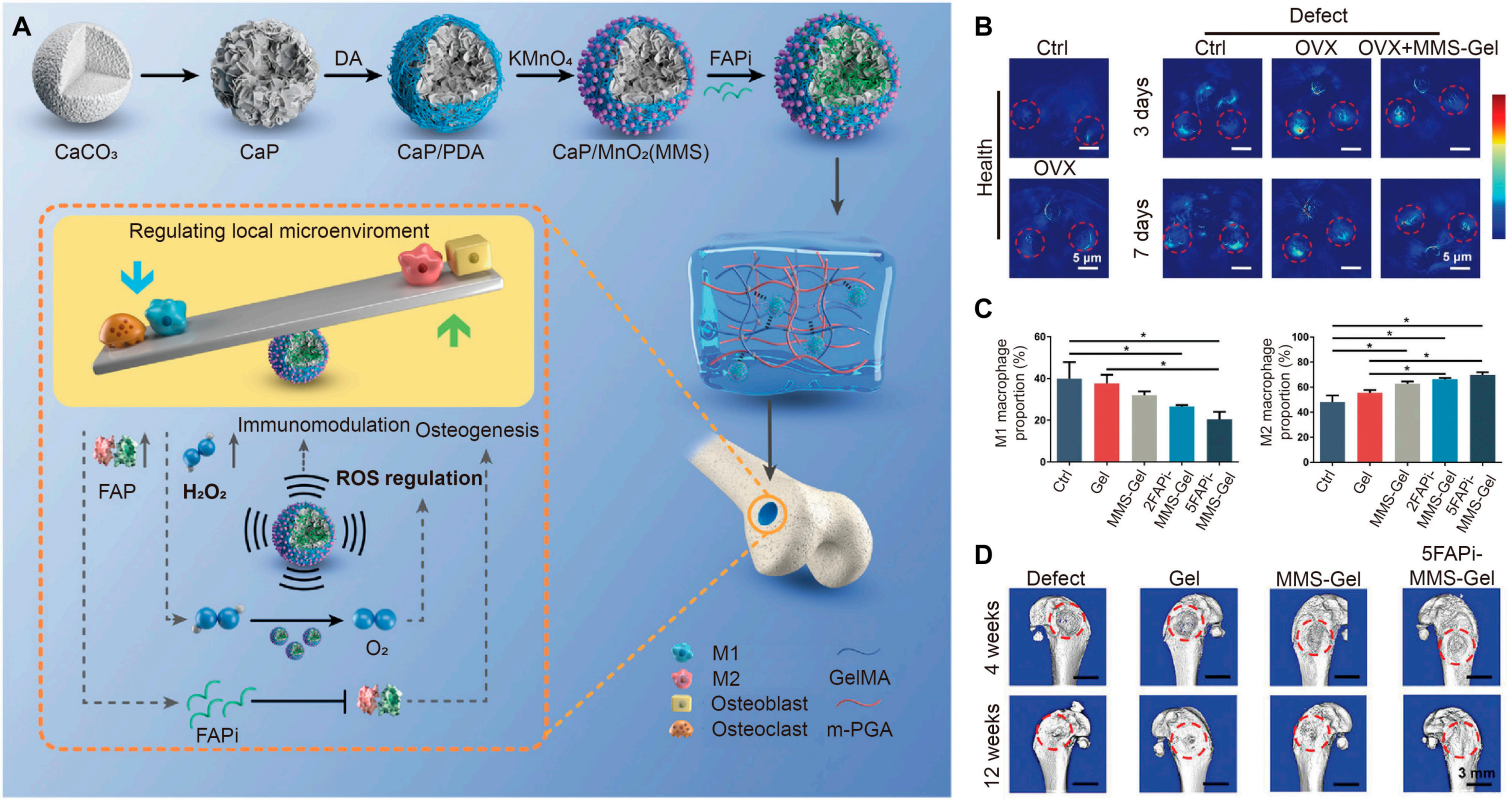
(A) Scheme of the preparation and effects of ROS-responsive hydrogels for osteoporotic bone defects. (B) ROS levels in bone defects. (C) Quantitative analysis of the proportion of M2 and M1 macrophages in bone defects. (D) Comparison of the microcomputed tomography (micro-CT) images of the different therapies for bone defects. Reproduced with permission from Ref. [88]. Copyright 2022 Wiley-VCH GmbH.

The body's most common oxidative agents, ROS, are produced in the mitochondria as a result of a partial decrease in oxygen during physiological processes [80]. ROS play a crucial role in numerous diseases, including osteoarthritis, osteoporosis, cardiovascular diseases, and infection [81,82]. The most prevalent members of the ROS family are hydrogen peroxides, hydrogen radicals, hydroxyl ions, and superoxide anion. ROS are key factors in bone homeostasis and bone remodeling processes [83]. It was shown that an increase in ROS levels in bone defects caused osteoclastogenesis, which prevented new bone formation [84,85]. In particular, the clinical treatment of osteoporotic bone defects remains difficult due to elevated levels of ROS and aberrant inflammatory responses. To address these issues, ROS-responsive hydrogels have been synthesized by introducing oxidization-labile linkers, such as thioketals, thioethers, and arylboronic esters. Additionally, nanoparticles with antioxidant properties, such as manganese dioxide (MnO_2_), ceria, and Prussian blue, are also employed in these hydrogels [86]. These ROS-responsive hydrogels scavenge ROS, relieve inflammation, and inhibit osteoclastogenesis and are an ideal material for bone tissue engineering, particularly for the treatment of osteoporosis [87]. For example, a multifunctional platform was developed by Chen et al. [88] to remove ROS and direct the immune response for osteoporotic bone defects (Fig. 3A). Fibroblast activating protein inhibitor (FAPi) loaded on MnO_2_-coated calcium phosphate microspheres reacted with hydrogen peroxides and functioned as a ROS-sensitive component. The hydrogels were created using methacrylated poly(glutamic acid) and methacrylated gelatin (GelMA) following UV irradiation in the presence of LAP, and then, the microspheres were inserted within hydrogels. Moreover, the ROS level in bone defects was significantly higher than that in healthy tissues in ovariectomized (OVX) rats. The ROS-responsive hydrogel reduced the ROS level in bone defects to alleviate the regenerative microenvironment (Fig. 3B). Additionally, calcium phosphate microspheres continuously released FAPi to control immunological responses. The composite hydrogel improved the percentage of M2 macrophages and reduced the M1 macrophage proportion (Fig. 3C). Additionally, the composited hydrogel scavenged ROS and released FAPi to modulate the microenvironment and promoted bone formation in the OVX rats (Fig. 3D). ROS-responsive hydrogels thus provide a promising strategy for the treatment of osteoporotic bone defects by targeting and modulating the microenvironment. However, the production of ROS varied with time in the defect sites, which may affect the sustained responsive performance of ROS-responsive hydrogels. Thus, dual- and multi-stimuli-responsive hydrogels could be designed to provide a stable response.

### pH-responsive hydrogels

pH-responsive hydrogels could respond to the change in pH values [89]. The pH of most healthy tissues is maintained between 6.5 and 7.2. However, the pH changes in some bone-related pathological processes, such as osteoporosis, bone defects, infection, and chronic inflammation [90]. In particular, an acidic local microenvironment (pH 4.0) created by mature osteoclasts adhering to the bone surface is crucial for bone resorption. Therefore, pH-responsive hydrogels have been designed for bone regeneration. These hydrogels have been induced through a number of functional groups, such as acetal, imine, hydrazone, oxime, and dimethyl maleate groups and MnO_2_ nanoparticles, that cause chemical reactions in an acidic or alkaline environment [91,92].

**Fig. 4.**
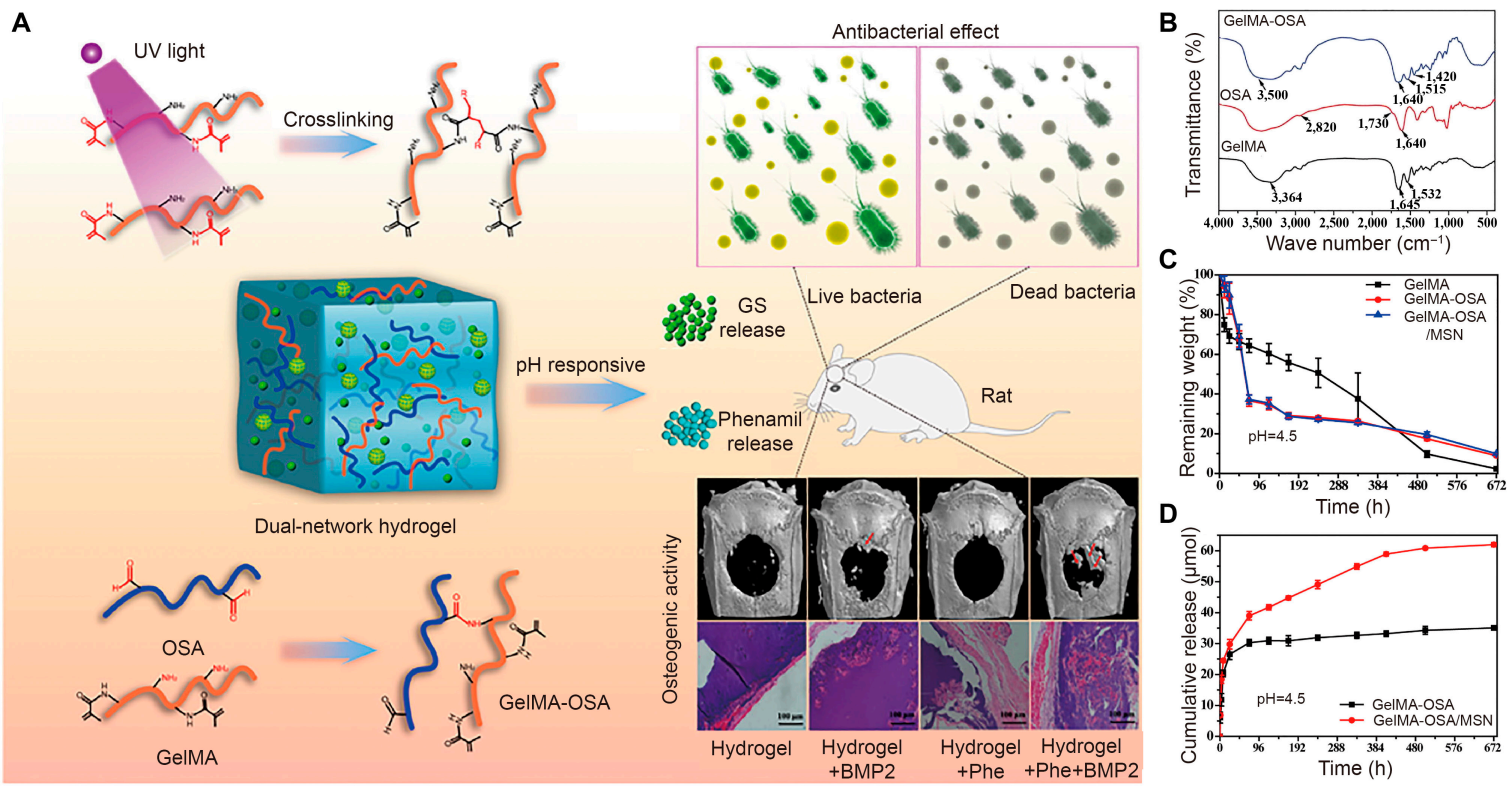
(A) Schematic diagram of the design and preparation of pH-responsive hydrogels for bone defect repair. (B) Fourier transform infrared spectroscopy (FTIR) spectra of GelMA, OSA, and GelMA-OSA. (C) Degradation rate of hydrogels at pH 4.5. (D) Drug released from hydrogels at pH 4.5. Reproduced with permission from Ref. [93]. Copyright 2021 Elsevier Inc.

The Schiff reaction is commonly found in pH-responsive hydrogels due to the generation of imine bonds. In this manner, the hydrogel can be formed under mild conditions. A pH-sensitive hydrogel containing the BMP-2 signaling activator phenamil and gentamicin sulfate (GS) was fabricated to improve the antibacterial effect and to promote bone regeneration (Fig. 4A) [93]. GelMA's amine groups and the oxidized sodium alginate's (OSA's) aldehyde groups were combined to create the Schiff-base linkages, which formed the main network of the pH-responsive hydrogel (Fig. 4B). Due to the photo-sensitive moiety of GelMA, a secondary network was formed under UV irradiation to enhance the mechanical properties. Due to the pH-responsive bonds, the degradation and release rates were accelerated under acidic conditions (Fig. 4C). To achieve the proper release rate, phenamil was loaded into mesoporous silica nanoparticles before mixing with the hydrogel, whereas GS was loaded directly into the hydrogel to release soon after hydrogel implantation (Fig. 4D). Thus, the pH-responsive hydrogel was formed at room temperature without other reagents and was degraded under acidic conditions. As hydrogels obtained by a Schiff base reaction lack mechanical strength and long-term stability, dual networks could be formed to overcome these problems. However, the pH of the implantation site cannot be predicted in the clinic and affects the surrounding tissue after implanting. Therefore, confirming the pH with devices before implanting and preparing dual-stimuli-responsive hydrogels that target other factors in the microenvironment of the disease could be considered.

## Physical Smart Hydrogels for Bone Regeneration

### Temperature-responsive hydrogels

**Fig. 5.**
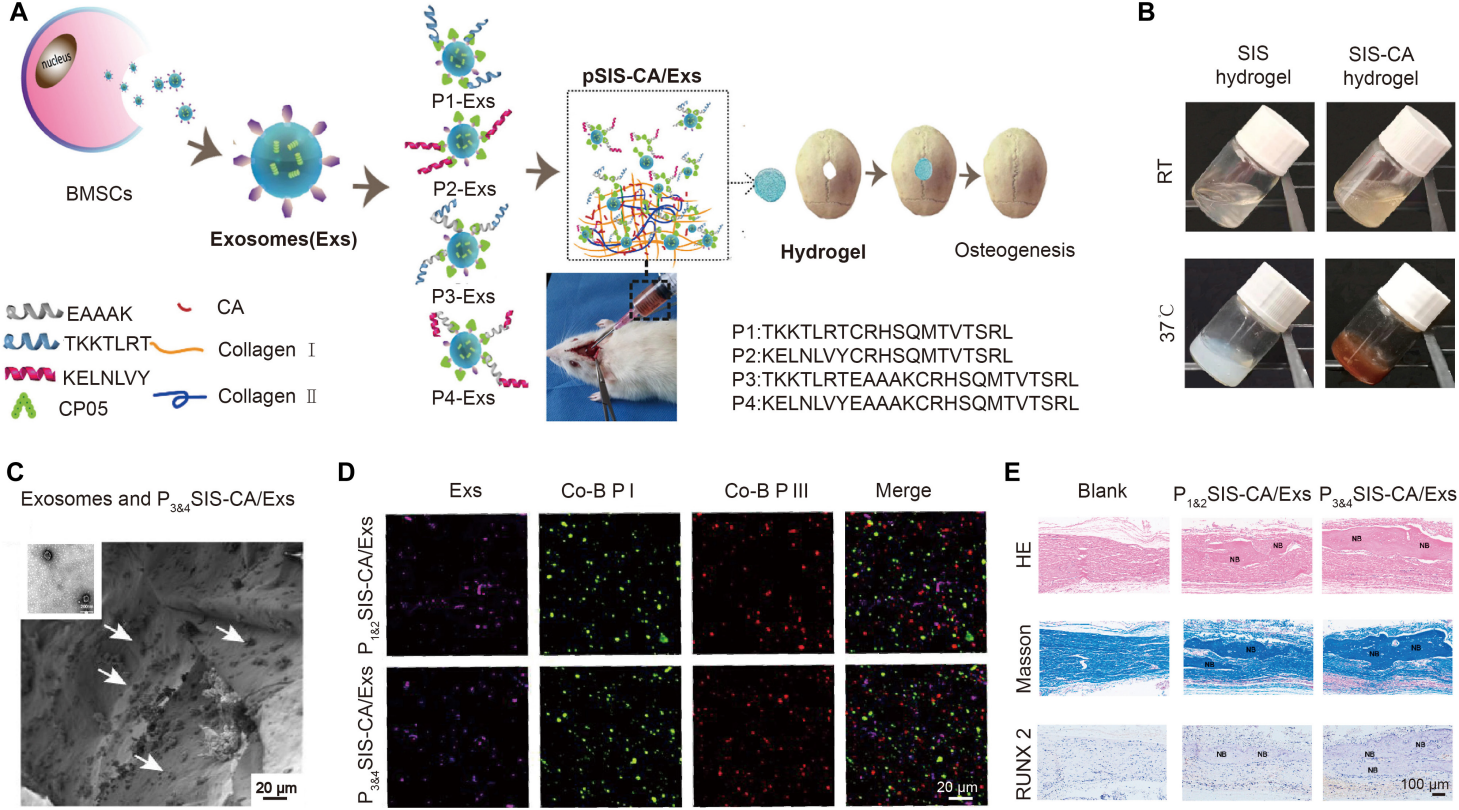
(A) Scheme for the preparation and application of thermo-responsive hydrogels. (B) Gelation of SIS and SIS-CA hydrogels. (C and D) Images of the exosomes anchored to hydrogels by peptides. (E) Comparison of new bone formation and expression of RUNX2 after different treatments in vivo. Reproduced with permission from Ref. [94]. Copyright 2021 Elsevier Ltd.

Thermo-responsive hydrogels have been studied for years, and they are widely used. The critical temperatures for a phase transition, known as the lower critical solution temperature and upper critical solution temperature, are 2 crucial properties of thermo-responsive hydrogels. The thermo-responsive hydrogels undergo a sol–gel phase transition while reaching the critical temperatures due to the hydrophobic interactions of polymers. Thermo-responsive hydrogels are commonly crosslinked via physical association under physiological conditions without additional molecules, and they are automatically formed in situ at bone defects. As a result, these hydrogels are convenient to use and can match the irregular shapes of bone defects. Ma et al. [94] designed an injectable thermo-sensitive hydrogel with exosomes and fusion peptides (Fig. 5A). Small intestinal submucosa (SIS) collagen molecules were employed to self-assemble into the hydrogel at 37 °C (Fig. 5B). The mechanical characteristics were improved by the addition of 3-(3,4-dihydroxyphenyl) propionic acid (CA). To efficiently load the exosomes, the hydrogels were submerged in a solution containing exosomes and fusion peptides (Fig. 5C and d). Through the activation of the phosphatidylinositol 3-kinase (PI3K)/protein kinase B (Akt) signaling pathway, the exosomes released from the hydrogels increased the expression of genes associated with osteogenesis (*RUNX2*, *ALP*, and *OPN*) and accelerated bone marrow mesenchymal stem cell (BMSC) development in the regenerative microenvironment, thereby promoting bone formation (Fig. 5E).

Although growth factor (GF)-loaded hydrogels have been explored as promising materials in repairing bone defects, it remains challenging to construct smart hydrogels with excellent gelation/mechanical properties as well as controllable GF releasing capability.

To achieve controlled release of growth factors, Lv et al. developed an injectable thermo-responsive hydrogel containing chitosan/silk fibroin, BMP-2-functionalized MgFe-layered double hydroxide nanosheets, and platelet-derived growth factor with two B subunits (PDGF-BB). Such a hydrogel could support stable sequestration of growth factors and achieve a sequential release of PDGF-BB and BMP-2 for efficient bone regeneration [48].

Clearly, thermo-responsive hydrogels undergoing sol–gel changes in response to temperature provide opportunities for efficient encapsulation and release of bioactive molecules in bone therapy. Moreover, the transformation of these hydrogels is reversible in the sol–gel phase according to temperature, which is convenient for noninvasive injection. Nevertheless, much attention should be paid during the operation and storage of thermo-responsive hydrogels due to their thermo-sensitivity to the surrounding temperature. To adapt to various biomedical uses, co-polymers and nanoparticles can be embedded in the hydrogels to modulate the responsive temperature.

### Mechano-responsive hydrogels

Mechano-responsive hydrogels deform under pressure from joint movements. These hydrogels usually possess excellent mechanical properties and repeatability. Bones experience various forces, and the behaviors of osteocytes are influenced by mechanical stimuli [95,96]. Physical cues could induce osteogenic differentiation of BMSCs [97]. Yu et al. [98] prepared mechanically robust hydrogels to promote osteogenesis by ten-eleven translocation 2 (Tet2) through the Tet2/HDAC1/E-cadherin/β-catenin pathway. Additionally, YAP/TAZ signaling was reported to be responsive to hydrogel stiffness [99,100]. Overall, it is important to consider mechanical signals while creating hydrogels [101,102]. The ability of hydrogels to reassemble after being physically damaged would be useful. Hou et al. [103] developed a self-integrating and shear-thinning hydrogel that possessed multiple hydrogen bond units of ureido-pyrimidinone on dextran polymers. During injection, the hydrogel exhibited shear-thinning behavior, behaving as a liquid under shear stress, and then immediately solidifying after injection. Hydrogel-containing chondrocytes, BMSCs, and BMP-2 were implanted subcutaneously in nude mice to create a cartilage–bone tissue complex, which exhibited an excellent osteogenic ability. The mechano-responsive hydrogel was dissociated by mechanical stress, but this could also be a feasible approach to release drugs on demand. For example, alginate-based hydrogels that contained PLGA nanoparticles continuously released drugs while joints moved [104]. The mechanically activated hydrogels have been fabricated for bone tissue engineering [105]. PLGA-based mechanically activated microcapsules (MAMCs) were embedded in polyethylene glycol diacrylate hydrogels. Dynamic compressive loading could rupture the MAMCs, which allows the drug to release and facilitate the healing of the musculoskeletal system.

Although several mechano-responsive hydrogels that demonstrated repeatability, deformation, and the ability to match the mechanical microenvironment have been developed for bone regeneration, there are still several challenges that need to be resolved before their use, such as the direction, intensity, and duration of mechanical stimulation. Thus, it is necessary to overcome these shortcomings and improve the strength of hydrogels to receive the stimulation. The mechano-responsive hydrogels have attracted considerable attention and have become a promising strategy for bone regeneration.

## Electromagnetic Radiation Smart Hydrogels for Bone Regeneration

### UV light-responsive hydrogels

Light can be employed as an ideal candidate trigger because it is spatiotemporally controllable, noninvasive, safe, simple to use, and contact-free [106]. As a result, many light-responsive hydrogels have been developed for use in biomedicine, such as scaffolds [107], wound dressing [108], and drug delivery systems [109]. By introducing photolabile components into the photo-responsive hydrogels, the hydrogels can form or degrade under light. Thus, the photo-responsive hydrogels are divided into photo-degradable hydrogels and photo-crosslinked hydrogels according to the functional groups. The main photoreactions in photo-responsive hydrogels are cleavage and addition. A number of photocleavable moieties, *ortho*-nitrobenzyl (*o*-NB), coumarin, and stilbene, have been widely used [110,111]. The addition reactions mainly include thiol-ene and cycloaddition.

**Fig. 6.**
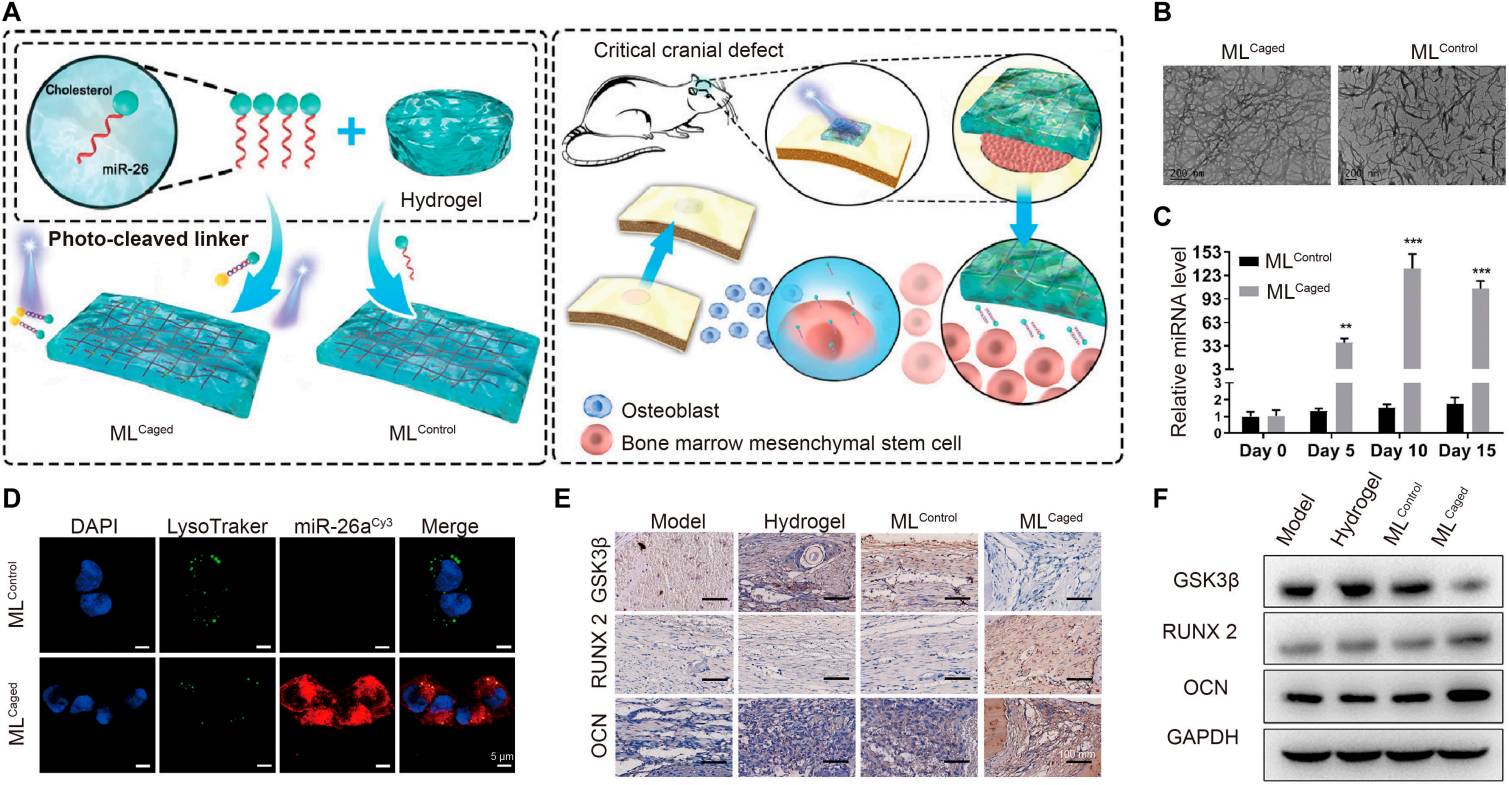
(A) Schematic illustration of miRNA-loaded UV light-responsive hydrogels for bone repair. (B) Images of ML^Caged^ and ML^Control^ hydrogels. (C) Release behaviors of exosomes in the hydrogels. (D) Images of exosomes taken up by hMSCs. (E and F) Expression of GSK3β, *RUNX2*, and *OCN* protein in the defect area. Reproduced with permission from Ref. [113]. Copyright 2021 Elsevier Ltd.

Recently, it has been demonstrated that microRNA-26 (miR-26) is a promising regulator for promoting osteogenesis, but the delivery and release system still limits its application [112]. To address this issue, an injectable UV-light sensitive hydrogel loaded with miR-26 was prepared (Fig. 6A) [113]. A photosensitive moiety containing the *o*-NB group was synthesized as a photo-cleavable linker to connect miR-26 with the PEG-based hydrogels via a Michael addition (Fig. 6B and D). miR-26 was released under UV light exposure in a continuous process, and the release rate depended on the irradiation time and intensity (Fig. 6C). Under light irradiation, the structure of *o*-nitrobester split into carboxylic acid and *o*-nitrosobenzyladehyde, breaking down the photosensitive groups [114]. Additionally, the levels of the osteogenic proteins *Runx2* and *OCN* were upregulated, and GSK3 protein was downregulated (Fig. 6E and F). After implantation, exosomes were released to enhance the osteogenesis of human mesenchymal stem cells (hMSCs) via 10-min UV irradiation at the bone defect sites. Thus, the light-responsive hydrogels released miRNA in a spatiotemporally controlled manner to modulate the bone regenerative microenvironment.

In biomedicine, photo-crosslinked hydrogels are commonly prepared by introducing light-responsive groups, such as acrylamide, norbornene, and methacryloyl [115]. The photo-crosslinked groups incorporated in the light-sensitive hydrogels enable the gelation process. Zhao et al. [116] prepared photo-crosslinked injectable hydrogel microspheres to capture Mg^2+^, which were formed by UV irradiation and which promoted bone formation at an osteoporotic bone defect. The GelMA was modified with bisphosphonate (BP) via a Schiff base reaction and an aldehyde activation reaction, and then, GelMA-BP microspheres were prepared to capture Mg^2+^ via a metal ion-coordination ligand by a microfluidic method. The hydrogel microspheres produced a sustained release of Mg^2+^ to activate osteoblasts and endothelial cells and inhibit osteoclasts by modulating the regenerative microenvironment. Therefore, the bone defects of OVX rats were repaired by the composited hydrogel microspheres.

As a result, UV light-responsive hydrogels have emerged as a promising candidate for bone regeneration, and they exhibit spatiotemporal controllability, noninvasiveness, and safety. UV as the trigger stimulated the degradation and gelation of the hydrogels. A handheld UV lamp is common, convenient, and inexpensive. However, their application may be challenged by the limited light penetration depth in tissues. Generally, the tissue penetration depth of UV light in healthy skin tissues is only 0.5 to 2.5 mm because of the light absorption and scattering by tissues [117]. To overcome this obstacle, the hydrogels are usually designed to be transparent. Furthermore, light-responsive hydrogels in response to near-infrared (NIR) light that can penetrate deeper into tissues have been developed as an alternative option.

### NIR-responsive hydrogels

NIR light has been widely used as an external stimulus in biomedicine due to its penetration capacity, nontoxicity, noninvasiveness, and maneuverability [118]. Compared with UV light, NIR can induce specific changes in responsive hydrogels and produces deeper tissue penetration [58]. NIR-responsive biomaterials in particular improved bone metabolism by activating pathways through enhanced oxidative metabolism in the mitochondria [119]. In addition, NIR leads to mild localized heat and a photoelectronic microenvironment that can induce osteogenic differentiation [120,121]. For example, an NIR-responsive hydrogel composed of methyl methacrylate, GelMA, and polydopamine nanoparticles was prepared using a free-radical polymerization method to study photothermal therapy for skull healing [122]. The composite hydrogels exhibited an excellent photothermal effect and were heated to 44.1 °C after NIR irradiation, and the hydrogels cooled when the NIR irradiation was removed. This phenomenon confirmed that the hydrogels exhibited NIR sensitivity and thermostability. Modulation of the photothermal microenvironment by NIR-responsive hydrogels accelerated bone regeneration.

**Fig. 7.**
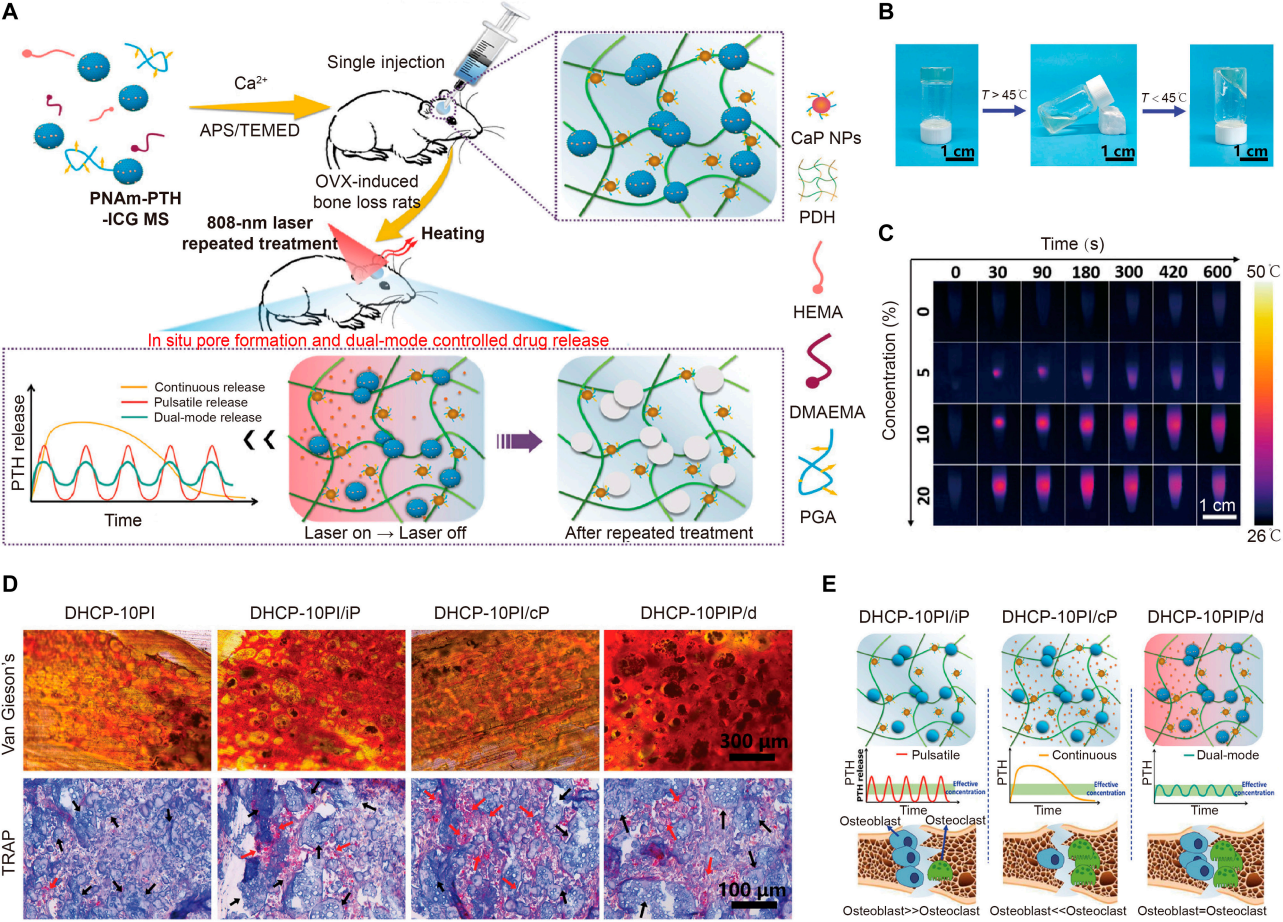
(A) Application of NIR-responsive hydrogels for osteoporotic bone regeneration. (B) Reversible sol–gel transformation property of the hydrogel. (C) Thermo-graphic images of NIR-responsive hydrogels using 808-nm NIR. (D) Histological observation of new bone formation in osteoporosis rats with different hydrogels. (E) Activities of osteoblasts and osteoclasts under different drug release patterns. Reproduced with permission from Ref. [123]. Copyright 2021 Wiley-VCH GmbH.

Additionally, NIR could be employed as a perfect trigger of a drug delivery system releasing drugs in a controlled spatiotemporal manner. Parathyroid hormone (PTH) has been used to treat osteoporosis in clinical practice due to its ability to activate both osteoblasts and osteoclasts. To control PTH release, Kuang et al. [123] developed a PTH-loaded calcium phosphate nanoparticle-coordinated poly(dimethylaminoethyl methacrylate-co-2-hydroxyethyl methacrylate) hydrogel for NIR-stimulated release to treat osteoporotic bone defects (Fig. 7A). The water-in-oil emulsion-based approach was used to create polymer microspheres comprising poly(*N*-acryloyl glycinamide-coacrylamide) (PNAm), indocyanine green (ICG), and PTH, which broke down and released PTH when exposed to NIR light, due to the photothermal impact of ICG. This smart hydrogel had a sol–gel phase transition at a temperature of 45 °C (Fig. 7B). Under NIR irradiation, the hydrogel demonstrated outstanding photothermal performance in vitro (Fig. 7C). The microspheres degraded and released PTH when the temperature rose to the PNAm phase transition point. The release modes (continuous release, pulsatile release, and dual-mode release) were managed by altering the NIR irradiation mode. The dual-mode groups that maintained PTH within a specific range showed the best osteogenic effectiveness in OVX rats, which may be related to the balance between osteoblast and osteoclast activities in the regenerative microenvironment (Fig. 7D and E). To avoid the burst release of BMP-2 in bone microenvironment, Wang et al. fabricated NIR-responsive polydopamine-coated microspheres as the carriers for BMP-2 delivery, and the microspheres were then incorporated into a thermo-responsive chitosan solution to form a dual-stimuli-responsive hydrogel. The polydopamine-decorated hydrogel allowed the conversion of NIR light energy into heat energy under exposure to NIR light, thereby allowing a controllable release of BMP-2 to enhance the osteoinductive effects [124].

Although NIR-responsive hydrogels are promising in bone repair, the NIR equipment may be expensive and difficult to operate. Moreover, the size of bone defects in the clinic may be too large for NIR irradiation, resulting in inconsistent behavior of hydrogels upon NIR stimulation. As a result, the range of NIR and potential for local overheating may restrict the application, and thermo-graphic images are required to monitor the range and temperature. Meanwhile, the equipment could be updated to extend the irradiation range.

### Magnetic-responsive hydrogels

Hydrogels that are sensitive to magnetic fields (MFs) exhibit responsive behaviors in a manner similar to NIR-responsive hydrogels. The MF-responsive hydrogels provide a number of benefits, including a rapid response, precise control, noninvasiveness, tissue penetrability, and a broad range [125]. Magnetically sensitive additives and polymer networks are often included in MF-responsive hydrogels. The hydrogel characteristics can be altered by magnetic-responsive nanoparticles by converting magnetic energy to heat or kinetic energy.

It has been shown that an MF induced osteogenic and chondrogenic differentiation of BMSCs to promote bone healing [126]. The effects may relate to the changes of the structure and orientation of the cell membrane and ECM proteins following treatment with MF. Therefore, various magnetic biomaterials, including iron oxide, transition metal ferrites, and transition metal alloys, have been developed and integrated into hydrogels to prepare MF-responsive hydrogels for bone regeneration. Iron oxide nanoparticles are one of the most popular magnetic biomaterials due to their biocompatibility and magnetic efficiency. MF-responsive hydrogels function in hyperthermia therapy as well as controlled release. To assess osteogenesis in a static magnetic field (SMF), Filippi et al. [127] constructed an MF-responsive hydrogel with magnetic nanoparticles (MNPs), a PEG-based network, and adipose-derived cells. The presence of thrombin-activated factor XIIIa enabled crosslinking of the factor XIIIa substrate-functionalized PEG polymers (PEG-Gln and PEG-MMP-Lys) to produce the hydrogel. MNPs were detected in the MF-responsive hydrogels using magnetic resonance imaging. Under SMF, the release of MNPs accelerated and then vanished after 7 days. Importantly, the MF-responsive hydrogel promoted osteogenic differentiation through the integrin, mitogen-activated protein kinase, and extracellular signal-regulated kinase pathways. Therefore, the MF-responsive hydrogel promoted vascularization and bone regeneration with MF exposure by releasing ions to regulate the regenerative microenvironment.

**Fig. 8.**
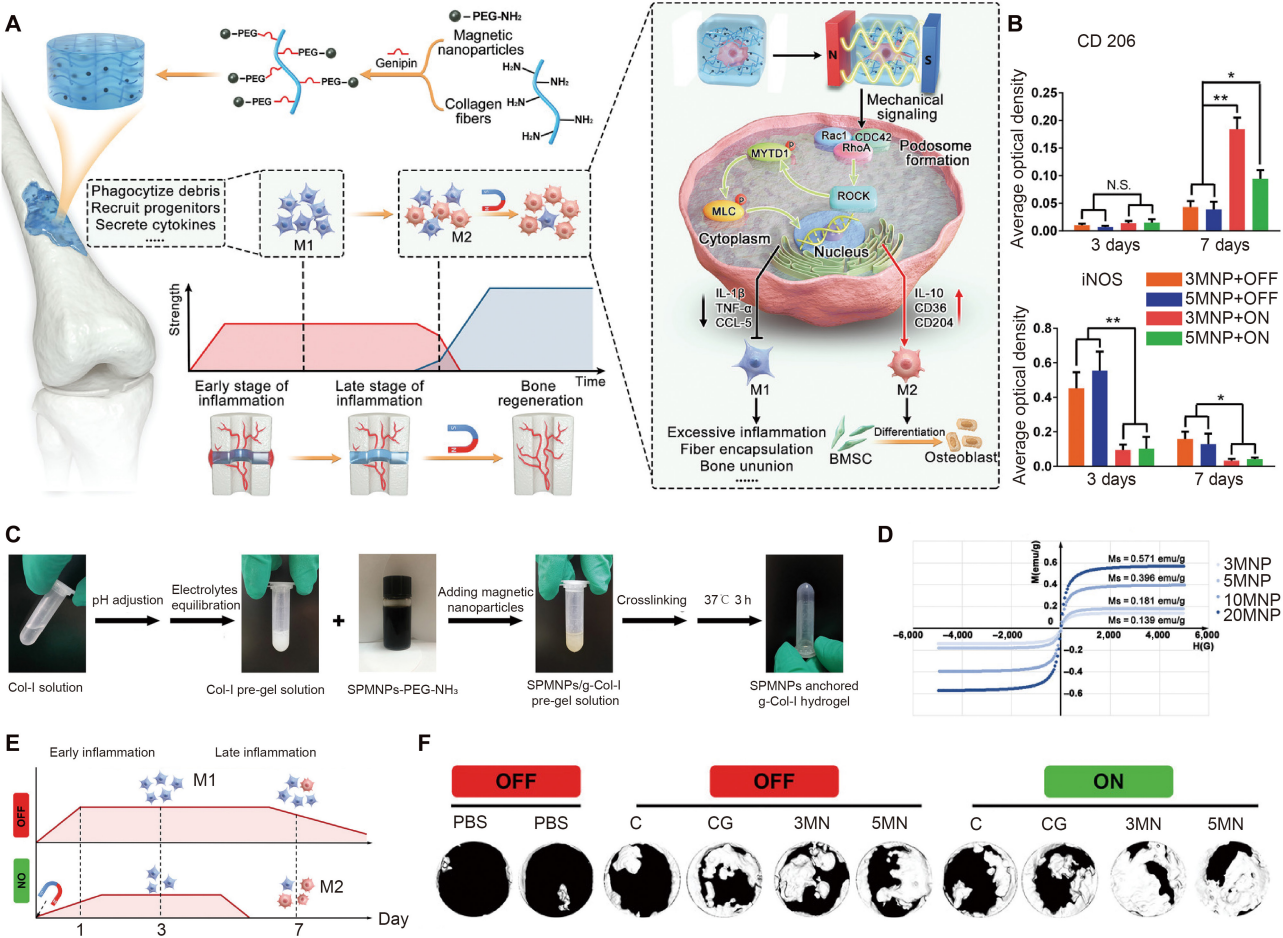
(A) Scheme of magnetic-responsive hydrogels modulating macrophage polarization with temporal control under a magnetic field to promote bone regeneration by activating the podosome/Rho/ROCK mechanical signaling pathway. (B) Quantitative analysis of the proportion of M2 and M1 macrophages after different therapies. (C) Synthesis and formation processes of MF-responsive hydrogels. (D) Magnetization curves of magnetic hydrogels with different concentrations of MNP. (E) Inflammatory process modulated by magnetic-responsive hydrogels under magnetic field exposure. (F) Comparison of the micro-CT images of different treatments for bone defects. Reproduced with permission from Ref. [129]. Copyright 2022 Wiley-VCH GmbH.

An MF can also regulate cell behaviors and improve the interactions between cells and the biomaterial. Recently, it was revealed that the balance between the proinflammatory phenotype (M1) and anti-inflammatory phenotype (M2) macrophages plays a vital role in tissue regeneration [128]. Therefore, the immune microenvironment has caught the attention of researchers. It would be ideal to modulate the macrophage polarization to M2 and alleviate inflammation. Huang et al. [129] prepared MF-responsive hydrogels to enhance bone repair by modulating the immune microenvironment (Fig. 8A and B). After being synthesized, the superparamagnetic nanoparticles were then grafted to collagen fibers using genipin that reacted with the free amino groups. The hydrogel was formed in 3 h at 37 °C (Fig. 8C). The hydrogel supermagnetic ability was asserted and then improved with an increased magnetic nanoparticle concentration (Fig. 8D). To preserve the essential role of M1 macrophages at the early stage of tissue healing, MF exposure was delayed for 1 week after surgery. Under an MF, the MF-responsive hydrogel network efficiently polarized encapsulated macrophages to the M2 phenotype via the podosome/Rho/ROCK mechanical pathway at the middle and late stages of tissue healing. Using a remotely scheduled approach, optimum immunomodulatory bone healing in vivo was finally achieved when macrophage polarization precisely matched the process of tissue regeneration (Fig. 8E and F). Overall, the MF-responsive hydrogels provided a remotely scheduled method for macrophage polarization, allowing for precise control of inflammatory development during tissue repair. In addition, MF can be used as a trigger to induce release of growth factors. Madani et al. developed a 2-compartment hydrogel consisting of an outer compartment of gelatin with stromal cell-derived factor 1-α (SDF-1α) and an inner compartment of alginate ferrogel with BMP-2. The release of BMP-2 from ferrogels could be controlled by MF at various time points. This system enabled the rapid recruitment of mouse mesenchymal stem cells (mMSCs) by SDF-1α and the delivery of BMP-2 in a delayed manner upon MF stimulation [130].

MF is a trigger with the advantages of spatiotemporal control, noninvasiveness, and safety, without requiring contact. Similar to NIR, MF can also enhance osteogenesis via a photothermal effect and immune microenvironment while providing deeper penetration and a broader effective range. The MF equipment and its parameters including range and intensity should be customized depending on bone defects, which, however, is often difficult to realize. The magnetic nanoparticles usually contain metallic elements, and thus, the long-term biosafety and cytotoxicity should be confirmed. To address this issue, it may be feasible to reduce the concentration of magnetic nanoparticles and select beneficial elements for biocompatibility.

### Electro-responsive hydrogels

**Fig. 9.**
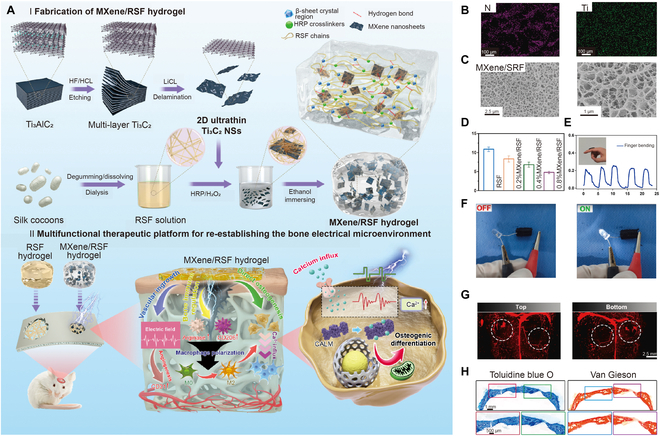
(A) Schematic diagram showing the fabrication process and osteogenesis effect of electro-responsive hydrogels. (B and C) Images of porous structures of the hydrogel. (D) Gelation times of the hydrogels with different MXene concentrations. (E and F) Conductivity of ES-responsive hydrogels after adding MXene. (G) Images of the 3D reconstruction of the blood vessels after the hydrogel implantation. (H) Histological images after the hydrogel implantation. Reproduced with permission from Ref. [52]. Copyright 2022 Elsevier B.V.

Electrical stimulation (ES), similar to other energy stimulations, can be controlled in a rapid and precise spatiotemporal manner. Electrical energy is common in the body, and it participates in physiological activities based on fundamental biological processes [131]. ES has been applied in the clinic for decades, particularly in bone healing. Related to this mechanism, ES affects transmembrane potentials and signaling of growth factors to modulate osteogenesis. In addition, ES induces cell proliferation, adhesion, and mineralization [132]. Since ES can promote osteogenesis, several electro-responsive hydrogels combined with conductive nanoparticles that regulate the biological electrical characteristics of the microenvironment have been developed for bone repair [133,134]. Hu et al. [52] constructed a silk fibroin-based conductive hydrogel encapsulating MXene nanosheets for bone healing, which contained a primary network crosslinked by HRP and H_2_O_2_ and a secondary physical network with a β-sheet structure (Fig. 9A to C). In this hydrogel, MXene nanosheets not only offered conductivity but also promoted the hydrogel formation due to the hydrogen bonding between MXene and the regenerated silk fibroin (Fig. 9D to F). This MXene and regenerated silk fibroin hydrogel also functioned as a piezoresistive pressure transducer, which tracked the electrophysiological microenvironment. With exogenous ES, the conductive hydrogels enhanced osteogenesis, increased M2 macrophage polarization, and promoted angiogenesis (Fig. 9G and H). Furthermore, the hydrogels enhanced osteogenic differentiation by activating the Ca^2+^/CALM signaling pathway. Therefore, electro-responsive hydrogels offer a unique and effective strategy for enhancing direct osteogenesis, regulating the immune microenvironment, and neovascularization for bone healing.

ES demonstrated osteogenic functionality, effective range modulation, and precise therapy. Nonetheless, external equipment is required. Moreover, since the electrodes need to be inserted into the defect area, it may cause some side effects, such as pain and infection. Therefore, the operation site should be sterilized, and the intensity and duration of ES should be confirmed and modulated to alleviate the pain.

## Dual/Multiple Smart Hydrogels for Bone Regeneration

Since the microenvironment of injured or diseased bone is accompanied by increased acidity, the inflammation and ROS levels are complicated, and a single smart hydrogel may be insufficient to achieve the goal of bone regeneration [135]. Therefore, dual- and multi-stimuli-responsive smart hydrogels have been developed to respond to various stimuli changes.

### Dual-stimuli-responsive hydrogels

**Fig. 10.**
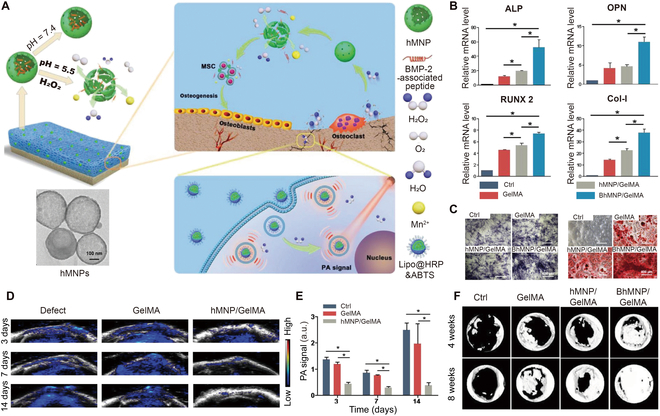
(A) Schematic diagram of a dual-stimuli-responsive hydrogel that scavenged H_2_O_2_ and promoted bone regeneration. (B) Expression of osteogenesis-related genes (*ALP*, *OPN*, *RUNX2*, and *COL-I*). (C) Comparison of images of osteogenesis after different treatments. (D and E) Images and quantitative analysis of ROS levels after hydrogel implantation in vivo. (F) Comparison of the micro-CT images after different hydrogels were implanted. Reproduced with permission from Ref. [136]. Copyright 2021 Wiley-VCH GmbH.

The injured bone microenvironment is complicated and usually contains excessive ROS under acidic conditions. The high level of ROS causes inflammation and osteoclastogenesis, while the acidic environment results in bone reabsorption and even osteoporosis. Therefore, regulation of both the acidic and ROS-rich aspects of the microenvironment is necessary for bone repair. Li et al. developed a pH- and ROS-responsive hydrogel consisting of GelMA and hollow MnO_2_ nanoparticles (hMNPs) loaded with BMP-2-associated peptides, and the composite hydrogel released oxygen and peptides on demand in response to an acidic and ROS-rich bone microenvironment (Fig. 10A) [136]. The nanoparticles encapsulating the peptides were broken down in the acidic environment, after which the hydrogel released BMP-2-associated peptides continuously for 28 days. Meanwhile, hMNPs decomposed H_2_O_2_ into H_2_O and oxygen. This helped to increase osteogenesis by alleviating the regenerative microenvironment (Fig. 10B and C). The antioxidant genes, such as silent information regulator type 1, superoxide dismutase 2, and catalase, were also enhanced to neutralize oxidative injury. ROS were depleted in the bone defect site after injecting the hydrogels (Fig. 10D and E). With modulation of the microenvironment, new bone formation and bone maturation were accelerated (Fig. 10F). Thus, the composite hydrogels not only released BMP-2-associated peptides on demand to promote osteogenic ability but also protected BMSCs from oxidative injury by eliminating ROS to modulate the local regenerative microenvironment.

### Multiple stimuli-responsive hydrogels

**Fig. 11.**
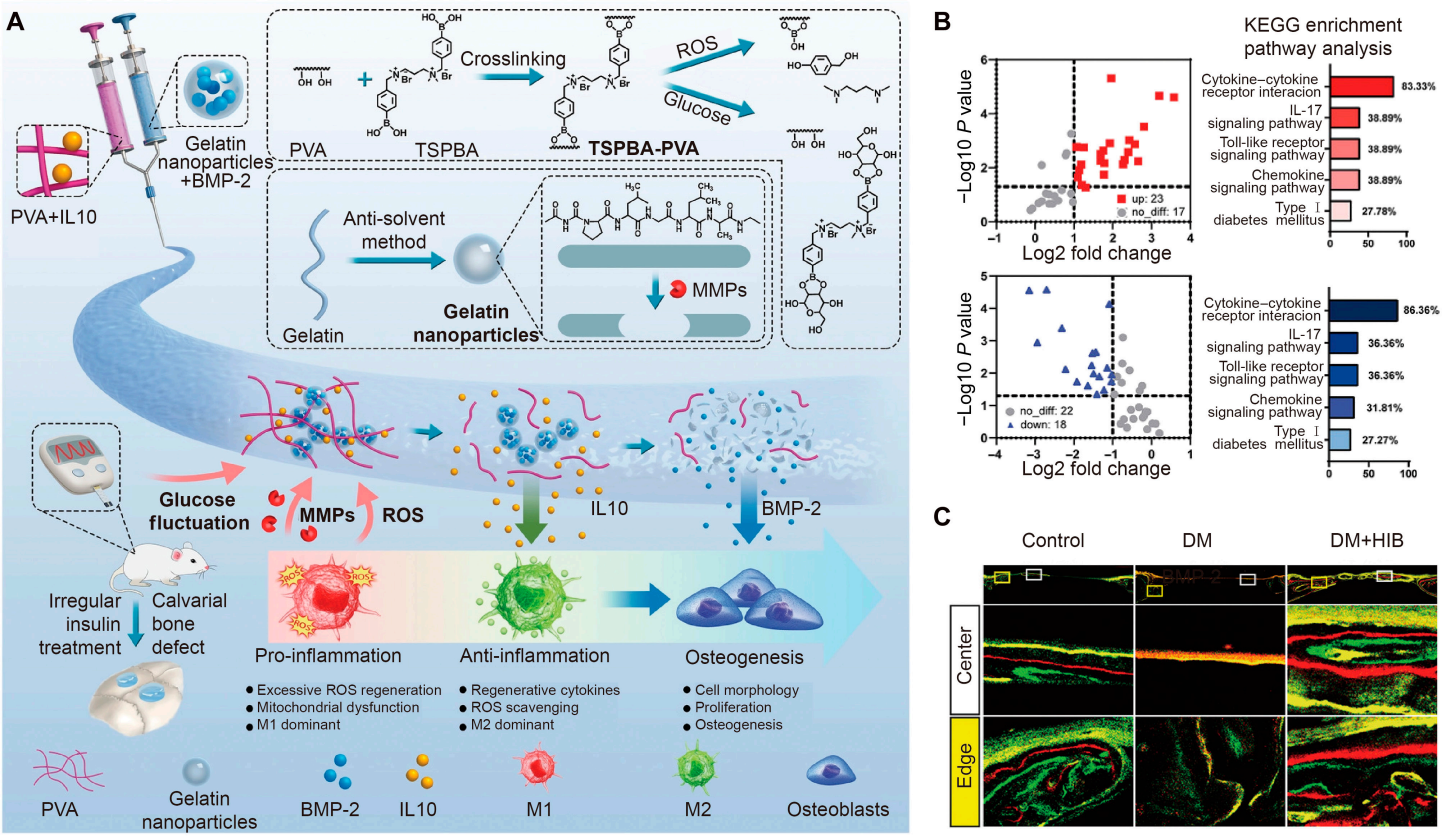
(A) Schematic diagram showing the multi-stimuli-responsive hydrogel for diabetic bone regeneration. (B) Volcano plot analysis and KEGG enrichment pathway analysis after different treatments. (C) Images of new mineral deposition in different groups. Reproduced with permission from Ref. [26]. Copyright 2022 Wiley-VCH GmbH.

It is more difficult for bone regeneration to occur in patients suffering from other diseases, particularly diabetes mellitus. Glucose fluctuations in diabetes induce metabolic disorder and mitochondrial dysfunction, which hinder tissue regeneration. Diabetic bone defects heal slowly due to high levels of inflammation and oxidative stress in the microenvironment. Therefore, multi-stimuli-responsive hydrogels can achieve multi-level activities when responding to the pathological bone microenvironment. To promote diabetic bone regeneration, Li et al. [26] developed a multi-stimuli-responsive hydrogel composed of a covalently crosslinked PVA and a colloidal network of gelatin nanoparticles (Fig. 11A). There is diagnostic logic in assessing multiple stimuli (glucose fluctuation, ROS, and MMPs) in the diabetic microenvironment and therapeutic logic for coordinating cargo delivery to coincide with the biocascade of inflammatory suppression and osteogenesis. A network of hydrogels was formed by phenylboronic acid (PBA)-based crosslinkers with the diol groups of PVA via phenylboronic ester linkages. Gelatin nanoparticles were formed by the cohesive interactions between amphoteric gelatin macromolecules. In this system, an interleukin 10 (IL-10)-loaded PVA network served as the backbone and was degraded by ROS and high glucose, while BMP-2-loaded gelatin nanoparticles provided sites for cell adhesion and were degraded by MMP. As a result, the hydrogels were responsive to multiple stimulations and released IL-10 and BMP-2 in order to regulate the pathological microenvironment. After hydrogel implantation, the immune cell function and mitochondrial function-related pathways play crucial roles in osteo-immunomodulation (Fig. 11B). The hydrogel regulated the macrophage polarization to alter cytokine production and consequently changed the microenvironment, with the inflammatory level downregulated via the related pathways. This smart hydrogel responded to 3 stimuli and achieved accurate drug delivery to ensure antioxidative homeostasis and match the immune-osteo progression, thus promoting diabetic bone repair (Fig. 11C). Therefore, multi-stimuli-responsive hydrogels stimulating a dynamic microenvironment provide a new strategy for treatment of pathological bone defects.

## Conclusion and Future Perspective

Rice et al. [137] showed that hydrogels combined with components from the regenerative microenvironment promoted the healing process, but these hydrogels were not smart. Smart hydrogels that are sensitive to the intra- and extracellular microenvironments have exhibited a variety of advantages in biomedical applications. Wei et al. [16] presented smart stimuli-responsive biomaterials for the treatment of bone defects combined with tumors, infections, or other bone diseases; however, they did not emphasize the role of the smart hydrogels. In this review, we emphasized the merits of smart hydrogels, summarized the hydrogel polymers, biochemical signals, physical stimuli, and electromagnetic energy for designing smart hydrogels with single-, dual-, and multi-stimuli responses for bone regeneration. Numerous polymers have been identified to prepare smart hydrogels that can be delicately inserted to meet multiple needs. The behaviors of hydrogels and their cargo can be altered under different stimuli, including degradation, gelation, deformation, ROS scavenging, nanoparticle transformation, cargo release, and oxygen production. These advanced designs contribute to the modulation of the regenerative and immune microenvironment. The regenerative and immune microenvironments play a vital role in bone regeneration, and smart hydrogels focused on microenvironment alterations to accelerate bone regeneration are a promising future strategy.

Water-containing biomaterials have received much attention for decades and have exhibited many advantages in academic research. Hydrogels have been widely used, such as in biological detection, disease treatment, and particularly in wound dressings. However, there are few hydrogel products that have been approved for bone regeneration. Considering the existing challenges in clinical translation, such as poor mechanical strength, biocompatibility, and biodegradability, substantial time is needed for smart hydrogels to transfer from the lab to clinic use. Future research is more likely to focus on the barriers before clinical translation, including the design of smart hydrogels, applications, clinical use, and commercialization.

1. Design and synthesis. The polymers, synthetic methods, and crosslinking reactions should be importantly considered in the design. Tailoring the properties of natural polymers limits the individual design. The development of polymer chemistry might provide future strategies for fabricating novel promising polymers. Another challenge is the crosslinking reaction. The slow rate in Schiff base reactions may affect cell distribution in the hydrogels, while the generation of free radicals via thiol-ene reactions may cause damage to cells. Due to its efficiency and safety in biological systems, especially in cell therapy, the bio-orthogonal reaction is recommended for use in smart hydrogel synthesis. Smart hydrogels in the future should be highly personalized and designed based on novel biological mechanisms and key targets or signaling pathways. Despite all these challenges in design and synthesis, functional group responsiveness to microenvironment alterations is an eternal topic. For example, mechanical properties are crucial for filling defects in weight-bearing areas, but local environment regulation is more concerned with specific conditions, such as diabetic fractures.

2. Hydrogel implantation is one of the most urgent needs in clinical applications. Local percutaneous injection is well-recognized due to its ease of operation and controllability, but the nonvisible subcutaneous tissue poses challenges for the accurate location of hydrogel injection in defect sites. Arthroscopic instruments and x-rays can be useful tools to assist the visualization. The matching rate of hydrogel degradation and bone formation is another critical challenge in this area, as the quality of newly formed bones can be affected by unbefitting degradation rates that are either too fast or too slow. Furthermore, the degradation rate is alterable in vivo and beyond our control. To address this point, functional constituents that either enable ex vivo detection or directly control the degradation rate are essential in future research. Clinical trials are necessary before clinical application, and there are numerous types of research on smart hydrogels for bone tissue engineering, but only a few related clinical trials are underway. We have found several smart hydrogels for bone healing on the Clinical Trials official website (www.clinicaltrials.gov). For example, a randomized controlled clinical trial (NCT05122299) evaluated the clinical effects of a coenzyme Q10 and collagen hydrogel, which was designed as a thermo-responsive hydrogel [138]. Although there were only 18 patients in the clinical trial, the process of clinic translation has begun.

3. Minimized complexity of the production process is critical in large-scale production. Sterilization is a necessary step after hydrogel production. The widely used sterilization methods in the clinic, such as heating, chemical disinfection, and irradiation, may be incompatible with smart hydrogels. Some sterilization technologies that are similar to the types of stimuli could induce gelation and degradation, destroy the structure of polymers, cause functional incapacitation, and generate by-products and therefore should be carefully applied or avoided. Hydrogel products are approved by regulatory bodies before commercialization, but some innovative materials may not be approved because of their inadequate evidence of safety. Alternative materials for smart hydrogel products should be further researched.

## Data Availability

The data are available from the corresponding author upon reasonable request.
